# Tactile acuity training for patients with chronic low back pain: a pilot randomised controlled trial

**DOI:** 10.1186/1471-2474-15-59

**Published:** 2014-02-26

**Authors:** Cormac Ryan, Nicholas Harland, Benjamin T Drew, Denis Martin

**Affiliations:** 1 Health and Social Care Institute, Teesside University, Middlesbrough TS1 3BA, UK; 2 Physiotherapy Department, Friarage Hospital, Northallerton DL6 1JG, UK

## Abstract

**Background:**

Chronic pain can disrupt the cortical representation of a painful body part. This disruption may play a role in maintaining the individual’s pain. Tactile acuity training has been used to normalise cortical representation and reduce pain in certain pain conditions. However, there is little evidence for the effectiveness of this intervention for chronic low back pain (CLBP). The primary aim of this study was to inform the development of a fully powered randomised controlled trial (RCT) by providing preliminary data on the effect of tactile acuity training on pain and function in individuals with CLBP. The secondary aim was to obtain qualitative feedback about the intervention.

**Methods:**

In this mixed-methods pilot RCT 15 individuals were randomised to either an intervention (tactile acuity training) or a placebo group (sham tactile acuity training). All participants received 3 sessions of acuity training (intervention or sham) from a physiotherapist and were requested to undertake daily acuity home training facilitated by an informal carer (friend/relative). All participants also received usual care physiotherapy. The primary outcome measures were pain (0-100visual analogue scale (VAS)) and function (Roland Morris Disability Questionnaire (RMDQ)). Participants and their informal carers were invited to a focus group to provide feedback on the intervention.

**Results:**

The placebo group improved by the greatest magnitude for both outcome measures, but there was no statistically significant difference (Mean difference (95%CI), p-value) between groups for change in pain (25.6 (-0.7 to 51.9), p = 0.056) or function (2.2 (-1.6 to 6.0), p = 0.237). Comparing the number of individuals achieving a minimally clinically significant improvement, the placebo group had better outcomes for pain with all participants achieving ≥30% improvement compared to only a third of the intervention group (6/6 vs. 3/9, p = 0.036). Qualitatively, participants reported that needing an informal carer was a considerable barrier to the home training component of the study.

**Conclusions:**

This pilot RCT found tactile acuity training to be no more effective than sham tactile acuity training for function and less effective for pain in individuals with CLBP. That the intervention could not be self-applied was a considerable barrier to its use.

**Trial registration:**

ISRCTN: ISRCTN98118082

## Background

Persistent pain has been associated with disrupted cortical representation of the painful body area within the somatosensory cortex of patients with complex regional pain syndrome [1,2] and phantom limb pain [3]. It has been proposed that this cortical disruption (or reorganisation) may play a role in pain maintenance and thus interventions aimed at normalising this disruption have been developed [4].

Sensory discrimination training, where the bodily area receives a stimulus and the patient must attempt to correctly identify key aspects of the stimulus (e.g. the precise location of the stimulus) is such an intervention. One of the first studies to use sensory discrimination training for patients with chronic pain was performed by Flor et al. [3]. In their study five phantom limb pain patients received electrical stimulation to eight adjacent but distinct parts of the residual limb (location) at different frequencies. Patients had to correctly identify the location and the frequency of the stimulation, and received feedback on their predictions from a therapist. Compared to a group of usual care controls, the sensory discrimination group had significantly greater improvements in pain and cortical reorganisation. Furthermore, the amount of improvement in pain, cortical reorganisation and skin sensory discrimination ability were all correlated with one another supporting the hypothesis that sensory discrimination training can improve the cortical representation of painful body areas and improve pain.

More recently, tactile acuity training, a form of sensory discrimination training, has been used to improve cortical representation in patients with chronic pain [5]. Tactile acuity training is an adaptation of the two point discrimination test, a measure of the minimum distance that can be detected between two points on the skin. The smaller the two point discrimination the better the tactile acuity. In tactile acuity training two probes (a narrow and a thick probe) are used to stimulate adjacent areas on the skin and patients must identify the location and mode (narrow/thick) of the stimulus, whilst receiving guided feedback from a therapist. Tactile acuity training can improve pain and two-point discrimination performance in patients with CRPS compared to tactile stimulation alone [5].

There is evidence to suggest that patients with CLBP have a distorted cortical representation of the sensorimotor area representing the lower back from studies directly measuring cortical representation using brain imaging [6], and studies indirectly measuring cortical representation using two-point discrimination [7,8], lumbopelvic motor control tests [9], laterality tests [10] and graphaesthesia [11]. Thus, it could be postulated that tactile acuity training may be beneficial for patients with CLBP.

A recent case series of patients with CLBP investigated the clinical effectiveness of a comprehensive sensorimotor retraining programme, a key component of which was tactile acuity training [12]. Following a 10 week intervention all three participants showed clinically meaningful improvements in pain intensity, pain interference and disability. A key limitation of this work was its case series design, which lacked an adequate control. A randomised controlled trial (RCT) of tactile acuity training for patients with CLBP is needed to build on this work and more robustly investigate the effectiveness of this intervention.

Previous investigations of tactile acuity training have included twice daily delivery of the intervention, once by a clinician in the clinical setting and once by an informal carer, (friend/relative) over a three week period [5]. This daily patient-therapist contact time would make the intervention impractical for use within the National Health Service (NHS) in the UK. Additionally, daily visits to the hospital setting would also place a significant burden on the patient. Self-management is an important component of the overall management strategy for people with long-term conditions and daily clinical visits may have a negative impact on a patient’s ability to self-manage, fostering feelings of dependency. Thus, in this pragmatic study the majority of the intervention was provided as part of a home training package. To comprehensively capture the experiences and perceptions of tactile acuity training of patients and their informal carer’s qualitative feedback was obtained via semi-structured focus groups. No such data exists within the literature and such information was considered important for the refinement of the intervention for future work.

The aim of this pilot, mixed-methods, RCT was to inform the development of a fully powered RCT by providing preliminary data on the effect of tactile acuity training for patients with CLBP compared to sham tactile acuity training for the primary outcome measures of pain and function. The secondary aim of this study was to obtain qualitative feedback from participants, via semi-structured focus groups, about their experiences and perceptions of the intervention to inform the refinement of the intervention and placebo intervention for a follow-up fully powered RCT.

## Methods

### Study overview

In this mixed-methods pilot RCT participants with CLBP were assigned to either a tactile stimulation (placebo) group or a tactile acuity (intervention) group. Participants were blinded as to which was the intervention group. Both groups attended three formal sessions (stimulation or acuity training) with a physiotherapist alongside a home training program to be facilitated by an informal carer (friend/relative). Additionally, both groups received usual care physiotherapy. Levels of pain and function were assessed pre and post treatment. The participants’ attitudes towards the treatment were captured in a focus group at the end of the study when all participants had completed their treatment. The trial was registered with the International Standard Randomised Controlled Trials Number (ISRCTN) and full details of the study can be found at http://www.controlled-trials.com/ISRCTN98118082.

### Setting and participants

This study took place in an NHS physiotherapy out-patient department within a UK hospital. Referrals to the department were screened by trained administrative staff and study invitations along with a participant information sheet and consent form were sent to all potentially suitable individuals with CLBP. Participants were recruited along with their individual informal carer (to deliver the home training program) by one of the research team (Drew). The inclusion criteria were:≥18 years of age; pain duration ≥6 months; non-specific CLBP with or without leg pain; no red flags indicating potential serious pathology; and no peripheral neuropathy. The exclusion criteria were: not having an informal carer (friend/relative) to assist with the home training programme; being unable to read English; and being unable to give informed consent. All participants provided written informed consent. The study received ethical approval from the National Research Ethics Service (NRES) Committee Northern & Yorkshire (Reference 11/NE/0328). Data were collected between January 2012 and July 2013. All participants who completed the study and their informal carers received a £20 high street voucher honorarium.

### Randomisation & blinding

Randomisation was performed by a member of the research team (Ryan) using a computerised random number generator and participants were assigned to either group using pre prepared concealed opaque envelopes. Participants were informed that there was a placebo group but they were unaware as to which group they had been assigned until after the study. It was not possible to blind the clinician (Drew), who also acted as the assessor.

### Sample size

This pilot study aimed to recruit 30 participants in total, 15 into each group. This number was based upon the recommendation that a pilot study for which there is no prior information upon which to base a sample size calculation should aim for 12 participants in each group [13].

### Interventions

#### Tactile acuity training (intervention)

There were two components to the tactile acuity training intervention: tactile acuity training and graphaesthesia acuity training. Tactile acuity training involved marking five/ten sites on the painful area. The sites were separated by the distance of the predetermined two point discrimination ability (see below for details). The sites were then stimulated in a random order using either a big (wine bottle cork) or small (pen top) probe, randomly applied [12]. Three blocks of 24 stimuli were performed in accordance with previous protocols [5]. The participant was required to concentrate on the stimulus saying which of the points was stimulated and which probe was used. If, after a number of sessions, the participant began to give the correct answers >90% of the time the marks were moved 10% closer to one another making the task more difficult. The tactile stimulation session was performed daily at home as part of the home training program and each session provided 72 stimuli over approximately 24 minutes. Graphaesthesia acuity training involved a series of 60 letters of the alphabet, approximately 1 inch high being traced on the painful area by the clinician (Drew) or informal carer using the tip of their finger [12]. The patient was asked to identify which letter had been traced on their back, and guided feedback was given. If they were correct they were informed of this. If they were incorrect the tester retraced the letter and informed the patient of the correct answer.

#### Tactile stimulation (Placebo)

The placebo group received the same tactile stimulation as the intervention group except that participants were not required to concentrate as they did not interact with the stimulus. Participants did not attempt to identify the details of the stimulus (location, probe size or letter) nor did the researcher/informal carer provide any feedback on the stimulus.

#### Treatment delivery

The first physiotherapy session was attended by the researcher and the participant. The informal carer was also invited to this first session. If the informal carer could not attend the 1^st^ session they were invited to attend one of the other therapist-patient sessions to observe the technique. The informal carer was shown how to fulfil the role of the tester for the purpose of the home training programme. The aim was for each participant to receive a minimum of 21 sessions (daily sessions for a minimum of three weeks). The maximum duration of the intervention was decided by the clinician providing usual care. The physiotherapist provided three sessions (intervention or placebo) while the informal carer was to provide all other sessions at home. Each participant was provided with a DVD demonstrating the technique (intervention or placebo as appropriate) as well as a simple instruction manual. At any point the participant and/or the informal carer could contact the physiotherapist for further assistance.

### Primary outcome measures

Pain and function were measured pre and post treatment. Pain was measured using the 0-100 mm Visual Analogue Scale (VAS) with 0 indicating no pain and 100 indicating pain as bad as it could be. Function was measured using the Roland Morris Disability Questionnaire (RMDQ). The RMDQ consists of 24 yes/no items and is scored on a 0-24 scale with higher scores indicative of greater functional limitations. Both of these outcomes measures have an established level of validity and reliability [14,15].

### Participant characteristics

A range of participant characteristics were measured including age, gender, height, weight, Body Mass Index (BMI), pain duration and baseline tactile acuity levels. Tactile acuity levels were measured using the two point discrimination test. The two point discrimination test was completed with all participants at the start of treatment to provide baseline levels of tactile acuity and to inform the distance between points to be used in the tactile acuity/stimulation training. Over the painful area a set of callipers was used to assess the smallest distance at which two points could be discriminated in line with the protocol of Wand et al. [8,11]. The callipers were set at 0 mm apart to begin and then increased in 2 mm increments. The increments continued until the participant noted that they could detect two points. Catch trials were included to prevent guessing. The callipers were then set at a value well above the initial identified threshold and the process repeated with descending increments. The threshold at which two points become one point was noted. Testing then continued between the two thresholds until a consistent response was ascertained for the minimal distance between two points that could be discriminated.

### Focus groups

When all treatment was completed, all participants (patient and informal carer) were invited to a focus group to explore their attitudes towards the intervention they received. There were two focus groups, one for the informal carers and one for the patients. The schedule for the semi-structured interviews used in both focus groups is shown in Additional file 1. The focus groups sought to gain insight into what aspects participants liked or disliked about the intervention along with any changes/improvements to the intervention they might suggest. The focus groups also asked about the home programme including how patients felt being facilitated by an informal carer and, conversely, how informal carers felt providing informal care.

### Methodology checking measures

#### Intervention credibility

Treatment credibility was assessed to see if participants in the placebo group found the training they received equally as believable as those in the intervention group in an attempt to verify appropriate participant blinding in keeping with the method of Moseley et al. [5]. Treatment credibility was measured using the 0-100 mm VAS with 0 indicating not credible and 100 indicating completely credible, with credibility defined as “how much did you believe in the treatment and think it might work”.

#### Home training assessment

On completion of the study participants were asked “how credible was home treatment compared to hospital treatment?” to see if participants found the home sessions equally as believable as the hospital based sessions. This was to evaluate if the home training component was of a similar standard to the physiotherapy delivered treatment. Again, treatment credibility was measured using the 0-100 mm VAS with 0 indicating not credible and 100 indicating completely credible. Participants were also asked to rate if they felt that the training performed by the informal carer was better, equally good, or not as good as the training delivered by the physiotherapist. Finally, participants and their informal carers were asked if they experienced any specific problems or issues with the home training programme (NO/YES), and if they answered yes they were provided with space to explain their answer.

To assess adherence to the home training programme all participants were asked to keep a diary of their home training activity.

### Data analysis

#### Quantitative data

Data normality was assessed using a 1 sample Kolmogorov-Smirnov test. All continuous data were found to be normally distributed and data were reported as mean (1SD). Nominal/ordinal data were presented as median (inter quartile range (IQR)) or mode. Change in pain and function from pre to post treatment, the primary outcome measures, were compared between groups using separate analyses of covariance (ANCOVAs), controlling for baseline values. Additionally, the number of individuals achieving a minimally clinically significant improvement, as defined by the Cochrane Back Review Group (30% improvement for pain VAS and 12% improvement for the RMDQ [16]), were compared between groups using a Mann-Whitney *U* test. Differences between groups for participant characteristics and methodological checks were performed using paired t-tests or the non-parametric equivalent. The analysis was by originally assigned groups.

#### Qualitative data

The focus groups were audio recorded and verbatim transcripts created. The transcripts were read and reread a number of times by one of the research team (Ryan) and emergent themes identified using thematic analysis. Identified themes were supported by direct quotes from participants.

## Results

### Participants

**Figure 1:**
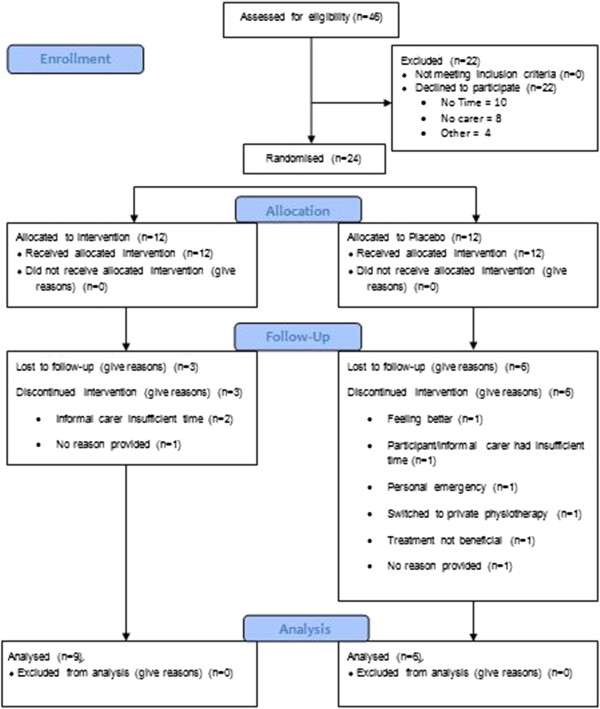
**Participant flow chart.** This figure shows the pathway of recruitment and retention of participants.

Twenty four individuals volunteered to participate for this study. Nine participants, three from the intervention group and six from the placebo group did not complete the study. Thus, 15 (9 intervention: 6 Placebo) participants completed the quantitative component of the study (Figure 1). Non-completers had a significantly shorter pain duration history than completers (10 vs. 3 years, p < 0.001) but there were no statistical differences for age, gender, height, weight, BMI or tactile acuity. There was no difference in participant demographics between the intervention group and the placebo group (Table 1). Eight participants (5 intervention; 3 Placebo) and their informal carers attended the focus groups.

**Table 1:** Participant characteristics

	**Intervention (n = 9)**	**Placebo (n = 6)**	**p-value**
**Age (years)**	45 (17)	46 (14)	0.92
**Height (m)**	1.69 (0.11)	1.64 (0.10)	0.36
**Weight (kg)**	79.3 (18.2)	73.6 (9.9)	0.50
**BMI (kg/m**^**-2**^**)**	27.8 (6.7)	27.4 (3.4)	0.90
**Tactile acuity (mm)**	61 (16)	57 (16)	0.63
**RMDQ (0-24)**	9.3 (6.6)	7.3 (3.1)	0.50
**Pain (0-100 mm)**	49 (19)	48 (31)	0.97
**Duration of symptoms (years)**	10.4 (13.5)	8.7 (11.4)	0.80

Legend: Data are presented as mean (1SD). BMI = Body Mass Index. RMDQ = Roland Morris Disability Questionnaire.

### Primary outcome measures

**Figure 2:**
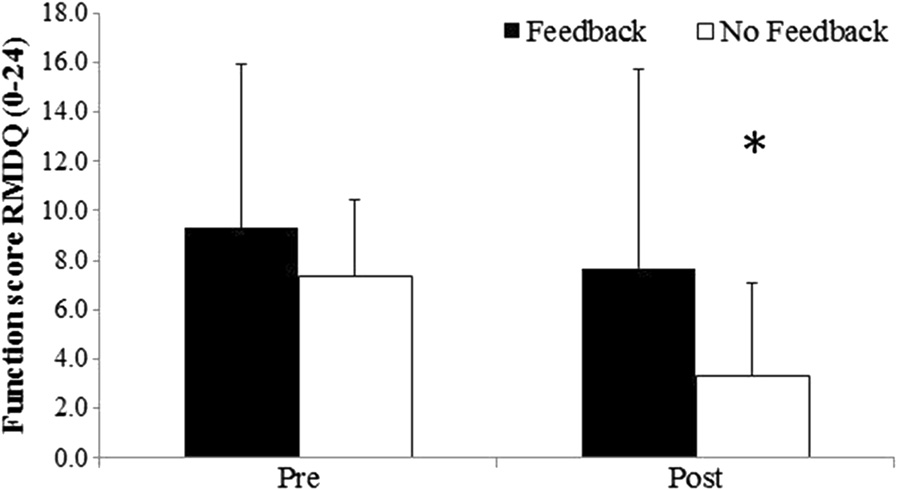
**Function levels pre and post treatment.** This figure shows the function levels, as measured by the Roland Morris Disability Questionnaire (RMDQ) pre and post treatment for both groups. * indicates a statistically significant (p < 0.05) change in function from pre to post treatment in the placebo group. Data are presented as mean (1SD).

**Figure 3:**
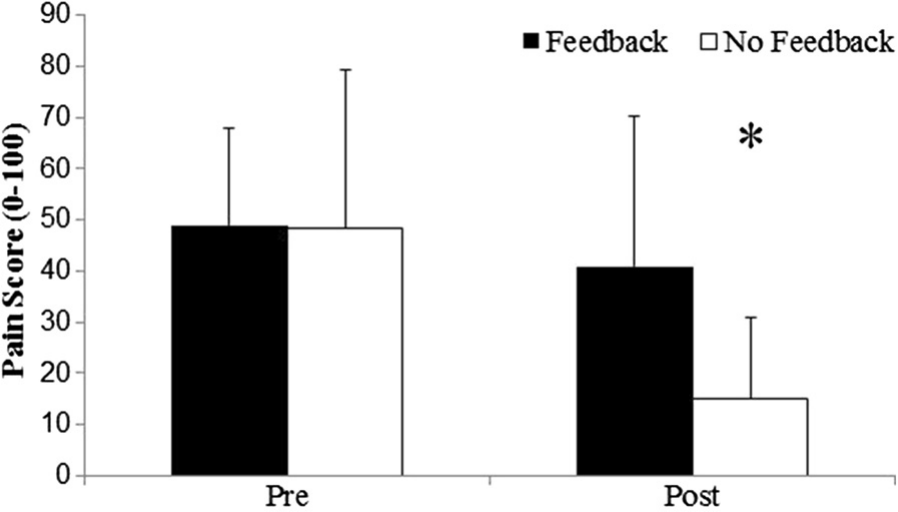
**Pain levels pre and post treatment.** This figure shows the pain levels, as measured by a 0-100 mm Visual Analogue Scale (VAS) pre and post treatment for both groups. * indicates a statistically significant (p < 0.05) change in pain from pre to post treatment in the placebo group. Data are presented as mean (1SD).

**Figure 4:**
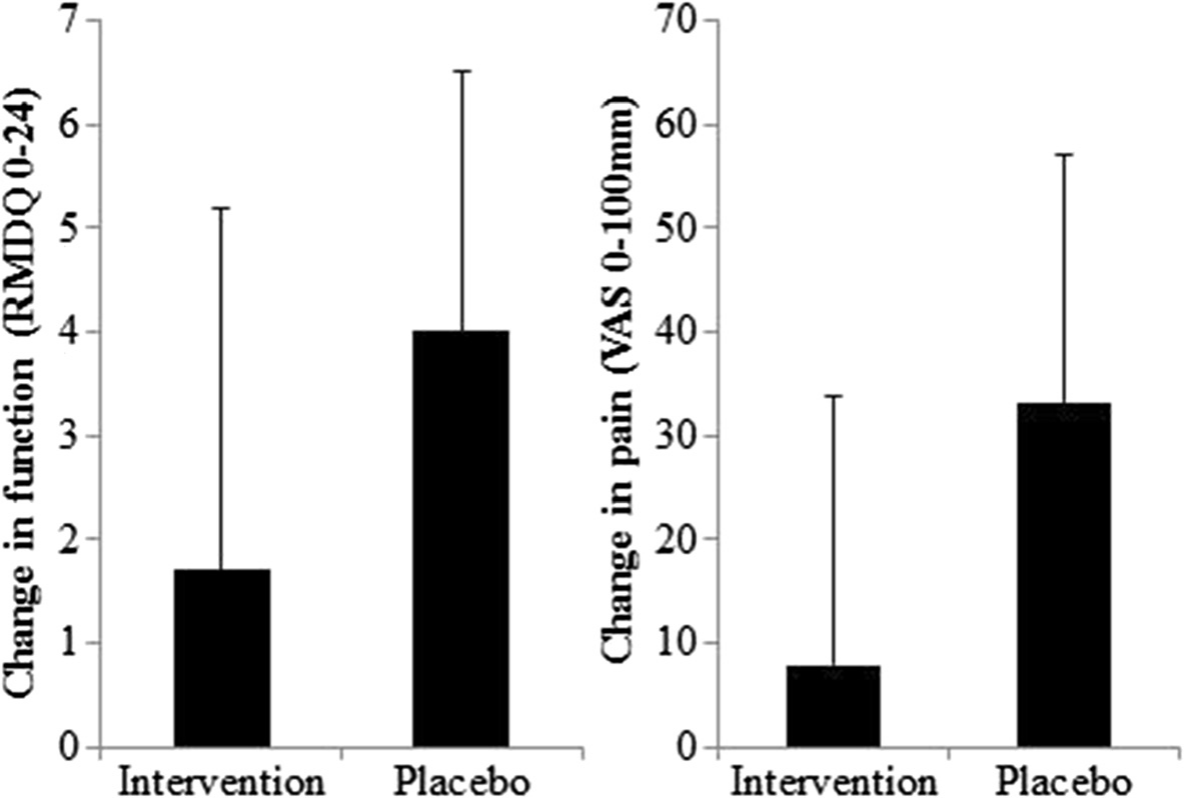
**Change in pain and function pre to post treatment.** This figure shows the change in pain and function levels from pre to post treatment, as measured by the 0-100 mm pain Visual Analogue Scale (VAS) and Roland Morris Disability Questionnaire (RMDQ) respectively. Data are presented as mean (1SD).

Pain decreased and function levels increased in both groups from pre to post treatment (Figures 2, 3, and 4). The magnitude of the change was greater for the placebo group, however, there was no significant difference (mean difference (95% CI), p-value) between groups for change in pain (25.6 (-0.7 to 51.9), p = 0.056) or function (2.2 (-1.6 to 6.0), p = 0.237) when controlling for baseline measures.

When the data were analysed by comparing the number of individuals achieving a minimally clinically significant improvement there was no difference between groups for function (4/9 vs. 4/6, p = 0.529). The placebo group, however, had significantly better outcomes for pain with all participants in the placebo group achieving 30% improvement or more compared to only a third of the intervention group (6/6 vs. 3/9, p = 0.036) (Table 2).

**Table 2:** Clinically significant improvement for individual participants

		**Function (RMDQ [0-24])**				**Pain (VAS 0-100 mm)**			
**Participant**	**Group**	**Baseline**	**Post**	**% change**	**≥12% improvement**	**Baseline**	**Post**	**% change**	**≥30% improvement**
1	I	3	2	33.3	Yes	50	50	0.0	No
2	I	22	23	-4.5	No	73	70	4.1	No
3	I	7	0	100.0	Yes	20	7	65.0	Yes
4	I	8	2	75.0	Yes	61	3	95.1	Yes
5	I	4	0	100.0	Yes	64	30	53.1	Yes
6	I	13	13	0.0	No	56	53	5.4	No
7	I	17	15	11.8	No	57	92	-61.4	No
8	I	4	4	0.0	No	18	18	0.0	No
9	I	6	10	-66.7	No	41	45	-12.2	No
10	P	8	3	62.5	Yes	55	18	67.3	Yes
11	P	9	1	88.9	Yes	88	11	87.5	Yes
12	P	6	5	16.7	No	13	5	61.5	Yes
13	P	3	0	100.0	Yes	22	0	100.0	Yes
14	P	12	10	16.7	No	80	45	43.8	Yes
15	P	6	1	83.3	Yes	32	12	62.5	Yes

*Legend: This table identifies which participants achieved a minimally clinically significant improvement for function and pain using the criteria of the Cochrane Back Review Group*[16]*. I = Intervention group, P = Placebo group, RMDQ = Roland Morris Disability Questionnaire, VAS = Visual Analogue Scale.*

### Methodology checking measures

The methodology checking measures are compared between groups in Table 3. There was no statistically significant difference between groups for any of these measures with both groups reporting a similar number of hospital based training sessions, usual care treatments, levels of treatment credibility and credibility of the home training component.

**Table 3:** Participant treatment details

	**Intervention (n = 9)**	**Placebo (n = 6)**	**p-value**
**Physiotherapist training sessions (number)**	2.8 (0.5)	3.0 (0.0)	0.17
**Home training sessions (number)**	36 (25)	39 (30)	0.83
**Usual care sessions (number)**	5.2 (2.3)	5.2 (1.7)	0.96
**Treatment credibility (0-100 mm)**	81 (19)	83 (19)	0.81
**Home vs. hospital training credibility (0-100 mm)**	67 (33)	77 (19)	0.52
**Home compared to hospital training**			0.63
***Hospital better (no.)***	2	1	
***Equal (no.)***	5	5	
***Home better (no.)***	0	0	

*Legend: Data are presented as mean (1SD) except for the home compared to hospital training outcome measure which reports participant numbers. Note for the number of physiotherapist and home training sessions variable, and the home* vs. *hospital training credibility variable data were provided by only 8 participants in the intervention group. For the home compared to hospital training variable data were only provided by 7 participants in the intervention group.*

### Focus group findings

Five themes were identified within the focus groups: 1. intervention barriers (with subthemes: practical, psychosocial and condition specific barriers): 2. scepticism and lack of understanding; 3. intervention effects; 4. concentration vs. relaxation; and 5. hospital better than home.

#### Intervention barriers

Participants identified multiple barriers to implementing the intervention. These barriers can be broken down into three subthemes: i. practical, ii. psychosocial and iii. condition specific barriers.

##### Practical barriers

Patients reported a number of practical barriers to carrying out the home training. These barriers included lack of time, difficulty co-ordinating their time with the informal carers time, the body markers washing away in the shower, difficulty lying flat for any period of time, and poor ergonomic set-up for the informal carers in the home environment. Additionally, participants felt the CD instructions and manual needed to be clearer. This was also consistently noted by the informal carers.

*“If one was in the other was out or* vice versa *so it was just trying to get two people, me and somebody else in the same room to do it” R7 (patient)*

“I don’t like the time that it took; it was a long time” R5 (patient)

“It was annoying having to write the numbers on every time you’ve had a shower remembering where the numbers stuck” R5 (patient)

“I can’t lie flat long enough for it to be done” R5 (patient)

“It wasn’t very good from my husband’s perspective to do it on the bed because he’s twisting his body in order to get to the areas so it wasn’t so good for him to do it on the bed because the bed’s not a very good height to bend over for that length of time and sitting on the bed he was twisting his body to do the treatment so that wasn’t good” R2 (patient)

“Well the CD we couldn’t even hear what he said” R3 (patient)

“The DVD wasn’t very good at all” R1 (informal carer)

There was also a barrier specific to the graphaesthesia training in that one of the participants reported her informal carer was dyslexic.

“my son is dyslexic and if he’s doing the b’s doing it the wrong way round” R4 (patient)

Another considerable difficulty noted by the participants was getting the informal carer to the hospital to observe the therapist undertaking the training/observation. For most this was to do with time but for one participant, who could not drive and required ambulance transportation, she was not allowed to be accompanied by an informal carer in the ambulance.

“I had to have the ambulance service bring me because I don’t drive and they don’t they wouldn’t let him [informal carer] come” R5 (patient)

##### Psychosocial barriers

Patients reported emotional/psychological barriers to the home training component, including feelings of embarrassment at being touched on the back by the informal carer and guilt at taking the informal carer’s time, despite patients not getting a sense that the carer shared these issues.

“He [informal carer/son] didn’t find it embarrassing but I did …… I was a bit embarrassed as well because it was my son who was doing it for me” R5

“It’s a big deal to me because it was quite time consuming for him as well and saying ‘do you mind if we do it now?’ I used to be quite apologetic you know having to say to him ‘do you mind just giving me fifteen minutes……..He didn’t mind doing it and never made a fuss about it, I just felt slightly guilty occasionally asking him” R4

In contrast to this some patients reported a great eagerness to help on behalf of the informal carer.

“I think the opposite [to guilt] really, I think Linda was so keen on this hoping that it could make a difference to me” R8

There was a sense that some of the guilt stemmed from a feeling that the treatment would not work and so asking the carer to help was a waste of their time.

“I just feel I wasn’t able to convince him that it would work, I really wanted to say right we can do this it will work and then I would have felt better about asking, not that he ever minded but I would have felt better” R4

In contrast none of the informal carers reported any emotional barriers associated with the home training.

##### Condition specific barriers

The participants noted some challenges to the intervention that were specific to their chronic back pain condition. One individual reported some paraesthesia/numbness in the area which made the testing difficult and frustrating. The same individual reported their pain increased during training because one area being stimulated was on a tender surgical scar, and it wasn’t until after a number of home training sessions that she asked her physiotherapist about this and he altered the positioning of the training away from the scar.

“The bits where I totally feel numb didn’t get them, I always them wrong all the time and I found it frustrating because he was going ‘oh no’ and I found it frustrating because the more I got it wrong” R5 (patient)

“at the really sensitive part I found that very painful, very painful and I hated the pin bit…” R5 (patient)

#### Scepticism and lack of understanding

Some participants did make indirect suggestions that they were not optimistic about the intervention having an effect and it seemed to be linked with a lack of understanding of the mechanisms of action of how the intervention might work.

“if it worked, we couldn’t see that it would but if it did” R8 (patient)

“I just feel I wasn’t able to convince him that it would work, I really wanted to say right we can do this it will work and then I would have felt better about asking, not that he ever minded but I would have felt better” R4 (patient)

#### Intervention effects

Some participants reported improved symptoms while others did not, with a participant from the placebo group reporting the greatest improvements. Two patients, both in the intervention group, did report improved or enhanced awareness of their lower back and some noted that this was maintained after the treatment had ceased.

“I found the area did improve, the sensitivity improved” R4 (Patient - intervention)

“Can feel it [my back] more…and actually having stopped .. the training the testing that area is still more sensitive than it ever has been for a long time.. knowing and feeling it” R2 (Patient-intervention)

#### Relaxation vs. concentration

During the training itself one of the participants reported that they felt relaxed and felt this might be due to the human touch component of the training and another reported being so relaxed during the treatment when doing it at night time that they were tending to drift off to sleep. In contrast to this one participant who stated that it could be relaxing also said that it required concentration and could be draining.

“It could be quite relaxing just being touched in that gentle kind of way” R4 (patient)

“I found that if I did it at night I feel asleep” R8 (patient)

“I found it really you had to concentrate…and I found it hard work, I was absolutely drained at the end of it every night” R4 (patient)

#### Hospital better than home

A number of the participants, when asked if the home delivery was better/worse than the hospital delivery, appeared to be in favour of the hospital setting. They stated that this was because the hospital setting was more *serious* facilitating better concentration on the training task, and they could get feedback/reassurance on whether the training was being carried out correctly and could ask questions.

“I think we take it more serious when we come to physio because when he’s doing it for you and when you’re at home it’s not as serious as; there’s a bit of messing about or you know….I think it was better coming doing it a physio because you’re concentrating more” R6 (patient)

“You could find out if there was anything you were doing wrong [with the hospital based sessions] R1 (patient)

## Discussion

The aim of this pilot, mixed-methods, RCT was to provide preliminary data on the effect of tactile acuity training for patients with CLBP compared to sham tactile acuity training for the primary outcome measures of pain and function. The secondary aim of this study was to obtain qualitative feedback from participants, via semi-structured focus groups, about their experiences and perceptions of the intervention.

The main finding of this study was that individuals in the placebo group had a statistically better outcome for pain than the intervention group, while there was no difference between groups for functional improvement. The main secondary finding was that there were considerable barriers to delivering the home training component associated with the need of having a second person (and informal carer) to deliver the training and the large amount of time the training required.

It is unclear why the placebo group had a better outcome for pain compared to the intervention group. One explanation may be related to relaxation. During the focus groups some participants suggested the training was relaxing whereas others suggested it was *hard work* and they felt *drained* after it. It is reasonable to assume that the intervention, for which concentration and feedback was of key importance, was mentally challenging and thus “draining” in comparison to the relatively relaxing placebo intervention, which required no concentration. There is evidence to suggest that relaxation can have a positive effect on pain outcomes [17,18] and this may, at least in part, explain the improved outcomes in the placebo group.

This is the first study to provide data on the effects of tactile acuity training for CLBP using an RCT design. The findings contrast with a recent case-series of three participants with CLBP which reported clinically important improvements in pain and function following tactile acuity training [12]. The reason for the contrasting findings may be related to the different methodologies used. In the case-series study, tactile acuity training was delivered as part of a comprehensive sensorimotor training package delivered over a minimum of 10 weeks. In the current study sensory discrimination training (tactile acuity training combined with graphaesthesia training) was used without a motor retraining component. Additionally, the case-series study had a comprehensive educational component and all participants were given the “Explain Pain” book. This may have helped patients to better understand the potential mechanisms of action and increased their expectancy, which may in turn have assisted with the quantity and quality of the training performed by the participants. In the current study the educational component explaining the mechanisms of action was relatively brief and simplistic. Without more detailed explanation patients may have been somewhat sceptical of the effects and thus may have had lower expectations. This is supported to some extent within the focus groups where patients implied a degree of scepticism about the intervention. Alternatively, the case-series design is more open to bias than the RCT design, which may explain the lack of effect in our study.

The findings of this study also contrast with those of Moseley et al. [5] who reported statistically significant effects of tactile acuity training compared to sham in patients with complex regional pain syndrome (CRPS). Again, the difference in findings may be due to differences in methodologies, primarily the difference in clinical sample. The evidence for cortical disruption in CRPS is compelling [19,20] and there is also evidence to show that as the cortical disruption declines so too does pain, though no cause and effect has been established [1,2]. The evidence to implicate cortical disruption in CLBP is reasonably strong [4,6] though perhaps less so than for CRPS. Furthermore, recent evidence suggests that somatosensory cortical disruption may be more associated with neuropathic type pain [21] which would explain why interventions targeting this disruption may be more beneficial for CRPS than CLBP.

In previous tactile acuity training studies [10,12] the intervention has been delivered on a daily basis by a therapist within a clinical setting. Such a treatment delivery system is not feasible within the current NHS. Thus the current study attempted to tailor the delivery system towards a predominantly home based non-therapist delivered system. Considering the lack of effectiveness shown in this study, adapting tactile acuity training in this manner to fit current clinical practice is not warranted. A recent prospective audit by Johnson et al. [22] investigated the effect of graded motor imagery (GMI) for CRPS in clinical practice. The GMI used was based on techniques which had proven successful under RCT conditions but had been adapted to fit with usual clinical practice (e.g. patient-therapist contact time was reduced). The audit found no effect of GMI on pain and highlighted the challenge of translating results of a complex pain intervention requiring a high level of patient compliance from the RCT setting into clinical practice. The results of the current study mirror these findings, in that tactile acuity training was adapted to fit within usual clinical practice delivery systems and this did not translate into an effective intervention. Studies such as ours and that of Johnson et al. [22] highlight the need for pragmatic trials to assess the clinical effectiveness of interventions to facilitate appropriate translation of research into practice. Furthermore, they emphasise the importance of developing interventions that can be easily applied in a typical healthcare setting.

### Study limitations

Perhaps the most significant limitation of this work was that no measure of tactile acuity or cortical disruption was taken post treatment. Thus we cannot be confident that tactile acuity training resulted in tactile acuity improvements, which would indirectly indicate an improvement in somatosensory cortical representation, the mechanism by which this intervention purports to work. It is worth mentioning that post treatment 2 point discrimination scores were collected for two participants both of whom were in the intervention group. For these participants two-point discrimination reduced from 51 mm to 50 mm and from 63 mm to 61 mm. The magnitude of change is minimal and questions the effectiveness of this intervention to bring about changes in tactile acuity and thus cortical representation. Only four participants in total (two from each group) were accompanied by their informal carer to observe the physiotherapist delivering the intervention. When this is considered alongside the qualitative feedback from participants that the training manual and DVD were difficult to follow it further highlights the significant barrier that needing a second person to deliver the home intervention creates. While our quantitative measure of credibility suggested that participants, in general, found the home and hospital based training to be similar, and similarly credible (see Table 2), this was limited in scope and possibly lacking precision. These issues would suggest that a future study should closely examine fidelity in more detail.

Another limitation of this study was its small sample size, which reduces the generalisability of the results and may have led to the study being underpowered, increasing the risk of a type II error. (Given the magnitude and direction of effect this is unlikely to be biased against showing a significant benefit of the intervention.) Of the 46 individuals who initially showed interest for this study and were screened 18 (~40%) declined to participate through lack of time or lack of an informal carer, further illustrating the practical challenge presented by the need for an informal carer to deliver the home training. Finally, an intention-to-treat analysis was not performed and only participants who provided data pre and post intervention were included in the analysis.

A key strength of this study was its mixed methods approach, which has identified a number of relevant barriers to the delivery of this intervention mostly around the need for a second person to provide the intervention. Of particular interest were the potential psychosocial barriers of guilt and embarrassment felt by some of the participants because of the need to involve an informal carer. These findings have potential implications not just for this intervention but within wider health care in areas where informal carers are required to deliver medical type interventions e.g. partners delivering routine subcutaneous injections in the home. The literature in this area is scant [23] and further investigation is warranted.

### Clinical implications and future studies

There were a number of practical clinical implications identified in this study, primarily around the barrier presented by needing a second person to deliver the home training. This barrier suggests that this intervention, in its current form, may not be practical within the UK’s NHS and some automated version of the intervention may be warranted. Barker et al. [24] investigated the effects of an automated sensory discrimination training device for patients with CLBP using the FairMed device. The device used vibrational stimulation rather than simple touch as used in tactile acuity training. The device was found to be similarly effective to TENS treatment although a high number of participants reported some kind of fault with the device (20/32), which may have negatively impacted on the treatment outcome when using the device. Automated devices such as the FairMed may be one approach by which sensory discrimination training techniques such as tactile acuity training may become more practical within the constraints of the NHS. Without dismissing the potential benefit of tactile acuity training, there is a need to investigate this intervention type using a more appropriate delivery system, as it may have been the latter which was responsible for the lack of effect of the active intervention in this study.

## Conclusions

In this pilot RCT there was no statistically significant difference between tactile acuity training and sham tactile acuity training for three of the four main outcome measures (pain and disability, both in absolute terms and as percentage change from baseline). Furthermore, the one significant change between the groups was in favour of the placebo group. These preliminary findings suggest that tactile acuity training as delivered in this study was not an effective adjunct treatment to usual care physiotherapy. However, considering the small pilot nature of this study, the questions around the fidelity of the home training component and the absence of a measure of tactile acuity post treatment, no firm clinical recommendations can be made. Participants reported a number of barriers to the home delivery of this intervention with the need for a second person being the primary issue. Future work should consider the need for an automated device to make home delivery more feasible, and the clinical effectiveness of such a device should be investigated.

## Competing interests

The authors declare that they have no competing interests.

## Authors’ contributions

CR conceived of the study, participated in its design and co-ordination, assisted with data collection, carried out the statistical/thematic analysis, contributed to the interpretation of the data and helped to draft the manuscript. BD was the primary collector of data, contributed to its interpretation, and helped to draft the manuscript. NH participated in the design and co-ordination of the study, assisted with data collection, contributed to the interpretation of the data and helped to draft the manuscript. DM participated in the design and co-ordination of the study, contributed to the interpretation of the data and helped to draft the manuscript. All authors read and approved the final manuscript.

## Pre-publication history

The pre-publication history for this paper can be accessed here:

http://www.biomedcentral.com/1471-2474/15/59/prepub

## Supplementary Material

Additional file 1 Focus group topic guide questions. Click here for file
