# Supplementary material for: Tactile acuity training for patients with chronic low back pain: a pilot randomised controlled trial
Source: BMC Musculoskelet Disord. 2014 Feb 26;15:59. doi: 10.1186/1471-2474-15-59 (PMC3942257; doi:10.1186/1471-2474-15-59)
Supplement: Additional file 1 — Focus group topic guide questions. [file 1471-2474-15-59-S1.doc]

**Additional file 1: Focus group topic guide questions:**

Participants with back pain focus group:

a) What were your feelings about the intervention?

b) What did you like about the intervention and what did you not like about the intervention?

c) How did you feel about being assisted with the home program by a friend/relative?

d) Do you feel that the home delivery was better/or worse than the physiotherapy delivery? and why?

e) What did you like or dislike about the home training component?

f) Do you think you would continue the home training component, yes or no? And why?

Informal Carer (Friend/relative) focus group:

a) What were your feelings about the intervention?

b) What did you like about the intervention and what did you not like about the intervention?

c) How did you feel about delivering the home program to your friend/relative?

d) Was there anything about it you liked or disliked?

e) Do you think you would continue the home training component if asked to do so by your friend/relative, yes or no? And why?
